# Supplementary material for: Efficacy and Immunogenicity of a Recombinant Vesicular Stomatitis Virus-Vectored Marburg Vaccine in Cynomolgus Macaques
Source: Viruses. 2024 Jul 24;16(8):1181. doi: 10.3390/v16081181 (PMC11359148; doi:10.3390/v16081181)
Supplement: Supplementary file 1 [file viruses-16-01181-s001.zip › viruses-3078016-supplementary.pdf]

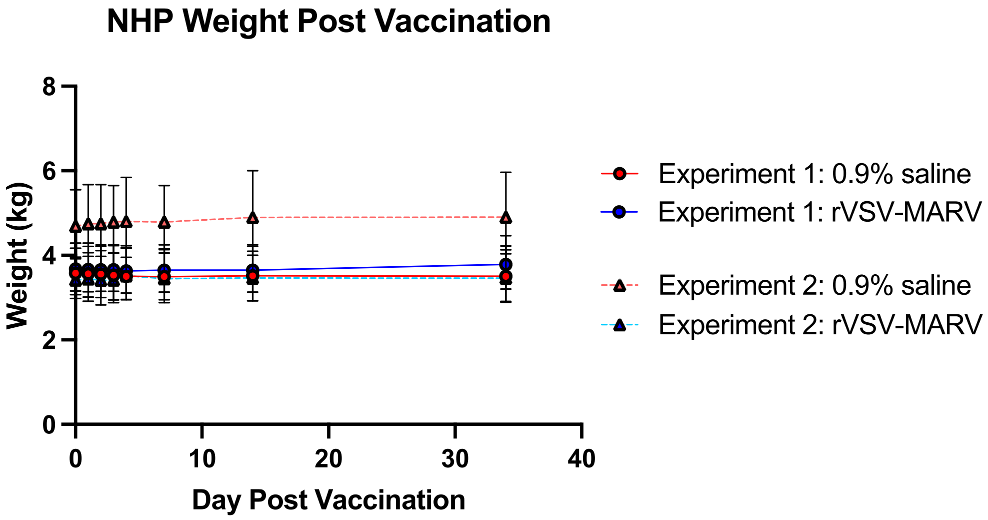

Figure S1. NHP Weights Post Vaccination. Experiments 1 and 2. Geometric means for groups are shown as circles for Experiment 1 and squares for Experiment 2. Groups given 0.9% saline are depicted in red/pink and groups vaccinated with rVSV-MARV in blue/light blue. Error bars show geometric standard deviation.

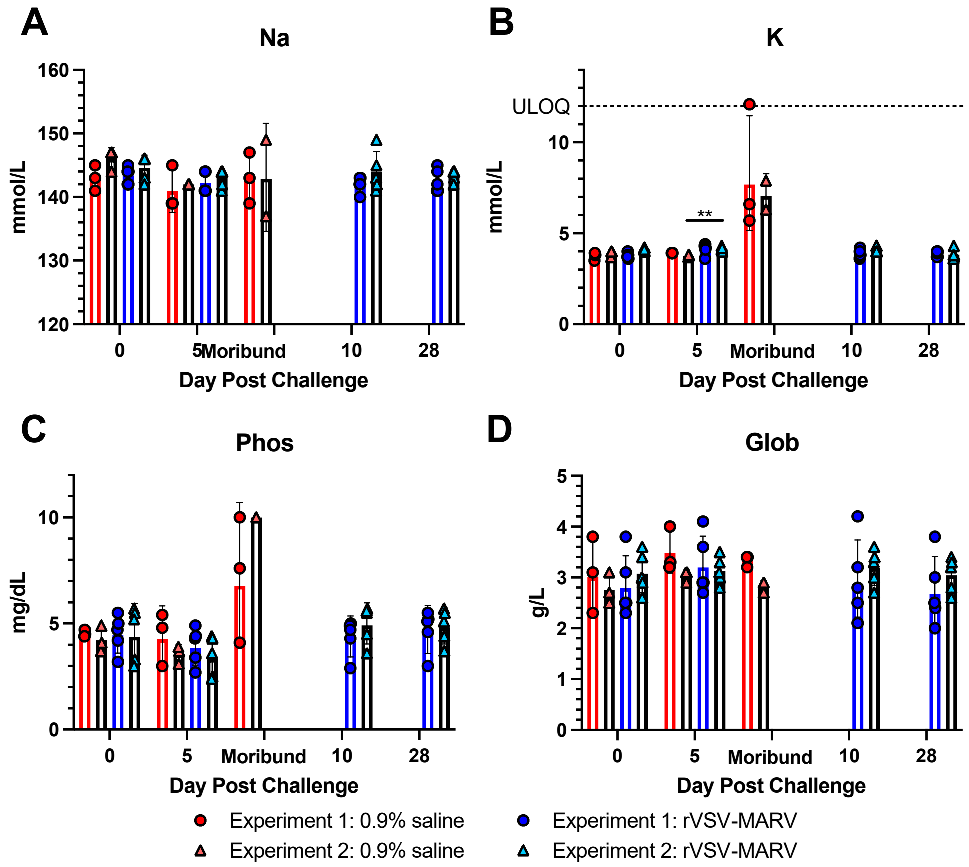

Figure S2. Other metabolite and electrolyte levels post MARV challenge. Levels of (A) Sodium [Na], (B) Potassium [K], (C) Phosphorus [Phos] and (D) globulin [Glob]. Red/pink circles show 0.9% saline-treated and blue/light blue triangles show rVSV-MARV-vaccinated NHPs. Solid red or blue bars denote Experiment 1, black bars Experiment 2 where the height is equal to the geometric mean of the group. The ULOQ for K is 12 mmol/L (horizontal dashed line) and one datapoint in the Experiment 1 0.9% saline group had a value >ULOQ, which is shown as 12.1 mmol/L. Error bars denote geometric standard deviation. A two-tailed unpaired t-test compared 0.9% saline and rVSV-MARV groups on a specific day (** p<0.01).

Day Post Vaccination	Group	Geomean/ SD	WBC (10^9/L)	LYM (10^9/L)	MON (10^9/L)	NEU (10^9/L)	EOS (10^9/L)	BAS (10^9/L)	RBC (10^12/L)	HBG (g/dL)	HCT %	MCV (fl)	MCH (pg)	MCHC (g/dL)
42 (0 dpc)	0.9% saline	Geomean	8.17	3.36	0.12	3.89	0.34	0.08	6.87	11.30	39.17	57.30	16.45	28.87
		Geo SD	1.33	1.30	1.43	1.85	2.20	1.78	1.10	1.01	1.02	1.11	1.11	1.01
	VSV-MARV vaccine	Geomean	6.95	3.33	0.21	4.28	0.52	0.08	6.39	11.90	39.50	61.92	18.67	30.19
		Geo SD	1.35	1.23	1.41	1.94	1.93	1.68	1.10	1.04	1.08	1.06	1.13	1.11
47 (5 dpc)	0.9% saline	Geomean	6.33	1.34	0.09	3.81	0.86	0.09	6.74	11.44	38.78	57.48	17.03	29.55
		Geo SD	1.15	1.43	1.37	1.30	1.56	1.16	1.07	1.04	1.04	1.10	1.11	1.01
	VSV-MARV vaccine	Geomean	8.88	3.22	0.13	4.07	0.87	0.13	6.59	12.11	40.56	62.10	18.37	29.83
		Geo SD	1.16	1.77	1.55	1.15	1.53	1.22	1.05	1.03	1.03	1.07	1.06	1.03
50 (8 dpc)	0.9% saline	Geomean	8.89	3.84	0.38	2.79	0.61	0.09	6.53	11.67	39.58	60.87	17.86	29.46
		Geo SD	2.26	1.10	1.16	5.86	3.00	3.84	1.02	1.18	1.12	1.10	1.15	1.05
	VSV-MARV vaccine	Geomean	NS	NS	NS	NS	NS	NS	NS	NS	NS	NS	NS	NS
		Geo SD												
52 (10 dpc)	0.9% saline	Geomean	NHP not on study	NHP not on study	NHP not on study	NHP not on study	NHP not on study	NHP not on study	NHP not on study	NHP not on study	NHP not on study	NHP not on study	NHP not on study	NHP not on study
		Geo SD												
	VSV-MARV vaccine	Geomean	7.56	3.33	0.15	3.06	0.77	0.11	6.03	11.58	37.23	62.10	19.29	31.24
		Geo SD	1.33	1.29	1.33	1.53	1.69	1.38	1.12	1.05	1.09	1.07	1.09	1.05
70 (28 dpc)	0.9% saline	Geomean	NHP not on study	NHP not on study	NHP not on study	NHP not on study	NHP not on study	NHP not on study	NHP not on study	NHP not on study	NHP not on study	NHP not on study	NHP not on study	NHP not on study
		Geo SD												
	VSV-MARV vaccine	Geomean	6.98	2.99	0.09	2.73	0.71	0.10	6.67	12.15	40.92	61.34	18.12	29.88
		Geo SD	1.34	1.17	1.41	1.57	2.15	1.38	1.07	1.04	1.04	1.05	1.06	1.02

Day Post Vaccination	Group	Geomean/ SD	RDWc %	RDWs (fl)	PLT (10^9/L)	MPV (fl)	PCT %	PDWc %	PDWs (fl)	LYM %	MON %	NEU %	EOS %	BAS %
42 (0 dpc)	0.9% saline	Geomean	18.06	39.32	256.97	8.88	0.23	36.53	13.92	41.06	1.47	47.62	4.09	0.92
		Geo SD	1.06	1.04	1.16	1.03	1.11	1.04	1.11	1.39	1.29	1.42	2.74	2.32
	VSV-MARV vaccine	Geomean	16.98	40.13	144.31	9.54	0.14	37.93	15.76	36.93	2.34	47.94	5.80	0.95
		Geo SD	1.03	1.03	1.56	1.06	1.46	1.05	1.18	1.44	1.72	1.54	1.61	1.34
47 (5 dpc)	0.9% saline	Geomean	18.17	39.81	167.52	8.99	0.15	38.55	16.36	21.25	1.33	59.53	13.44	1.50
		Geo SD	1.08	1.02	1.27	1.10	1.17	1.05	1.22	1.58	1.61	1.20	1.44	1.00
	VSV-MARV vaccine	Geomean	16.91	39.91	227.97	9.83	0.22	38.62	16.84	36.93	1.39	45.85	9.89	1.50
		Geo SD	1.03	1.03	1.18	1.08	1.13	1.07	1.22	1.58	1.36	1.29	1.57	1.00
50 (8 dpc)	0.9% saline	Geomean	18.28	42.79	146.90	10.16	0.15	40.27	18.39	42.97	4.21	31.37	6.83	1.02
		Geo SD	1.06	1.03	1.65	1.08	1.77	1.01	1.04	2.03	1.93	2.59	1.34	1.71
	VSV-MARV vaccine	Geomean	NS	NS	NS	NS	NS	NS	NS	NS	NS	NS	NS	NS
		Geo SD												
52 (10 dpc)	0.9% saline	Geomean	NHP not on study	NHP not on study	NHP not on study	NHP not on study	NHP not on study	NHP not on study	NHP not on study	NHP not on study	NHP not on study	NHP not on study	NHP not on study	NHP not on study
		Geo SD												
	VSV-MARV vaccine	Geomean	16.96	40.13	205.75	9.52	0.20	37.46	15.47	43.91	2.03	40.48	10.16	1.43
		Geo SD	1.03	0.00	1.43	1.10	1.35	1.06	1.21	1.23	1.20	1.23	1.44	1.10
70 (28 dpc)	0.9% saline	Geomean	NHP not on study	NHP not on study	NHP not on study	NHP not on study	NHP not on study	NHP not on study	NHP not on study	NHP not on study	NHP not on study	NHP not on study	NHP not on study	NHP not on study
		Geo SD												
	VSV-MARV vaccine	Geomean	17.05	40.12	169.14	10.08	0.17	38.72	16.30	44.59	1.22	39.40	10.77	1.43
		Geo SD	1.02	1.03	1.37	1.05	1.32	1.05	1.17	1.31	1.41	1.19	1.62	1.10

Table S1. Summary statistics for CBC Experiment 1. Day 50/ 8dpc samples were from NHPs that were euthanized or found dead. NHP not on study=NHP deceased. NS- no sample run. Abbreviations: Total white blood cell count (WBC), total lymphocyte cell count (LYM), total monocyte cell count (MON), total neutrophil cell count (NEU), total eosinophil cell count (EOS), total basophil cell count (BAS), total red blood cell count (RBC), total hemoglobin (HGB), hematocrit value (HCT), mean cell volume (MCV), mean corpuscular hemoglobin (MCHC), platelet count (PLT), mean platelet volume (MPV), plateletcrit (PCT), platelet distribution width (PDWc and PDWs), red cell distribution width (RDW), percent neutrophils (NEU%), percent lymphocytes (LYM%), precent monocytes (MON%), percent eosinophils (EOS%), percent basophils (BAS%).

Day Post Vaccination	Group	Mean/SD	WBC (10^9/L)	LYM (10^9/L)	MON (10^9/L)	NEU (10^9/L)	EOS (10^9/L)	BAS (10^9/L)	RBC (10^12/L)	HBG (g/dL)	HCT %	MCV (fl)	MCH (pg)	MCHC (g/dL)
42 (0 dpc)	0.9% saline	Geo Mean	7.30	3.12	0.06	3.11	0.81	0.11	6.82	12.45	41.37	60.66	18.31	30.11
		Geo SD	1.05	1.24	1.31	1.23	1.03	1.06	1.02	1.02	1.01	1.01	1.02	1.01
	VSV-MARV vaccine	Geo Mean	8.28	3.35	0.14	3.38	0.79	0.11	6.00	11.66	37.09	61.62	19.42	31.43
		Geo SD	1.42	1.36	2.06	2.07	1.60	1.53	1.09	1.04	1.09	1.06	1.10	1.08
47 (5 dpc)	0.9% saline	Geo Mean	4.60	1.12	0.05	2.46	0.84	0.07	6.41	12.04	38.96	60.66	18.77	30.86
		Geo SD	1.55	1.25	1.27	1.76	1.58	1.54	1.04	1.04	1.04	1.01	1.02	1.03
	VSV-MARV vaccine	Geo Mean	8.47	3.58	0.10	3.44	0.98	0.12	6.18	11.70	38.38	61.91	18.90	30.44
		Geo SD	1.38	1.29	1.87	1.66	1.69	1.39	1.05	1.05	1.04	1.06	1.06	1.01
49 (7dpc, M)	0.9% saline	Geo Mean	7.13	4.39	0.37	1.14	1.12	0.11	6.37	12.83	40.06	63.00	20.13	31.97
		Geo SD	1.00	1.00	1.00	1.00	1.00	1.00	1.00	1.00	1.00	1.00	1.00	1.00
50 (8 dpc, M)	0.9% saline	Geo Mean	28.07	7.85	0.24	17.70	1.81	0.42	5.61	11.23	35.33	63.00	20.10	31.93
		Geo SD	1.00	1.00	1.00	1.00	1.00	1.00	1.00	1.00	1.00	1.00	1.00	1.00
52 (10 dpc)	0.9% saline	Geo Mean / Geo SD	N/A	N/A	N/A	N/A	N/A	N/A	N/A	N/A	N/A	N/A	N/A	N/A
	VSV-MARV vaccine	Geo Mean	6.94	3.42	0.09	2.07	1.02	0.11	6.10	11.53	37.91	61.91	18.91	30.43
		Geo SD	1.50	1.43	1.63	1.91	1.94	1.47	1.04	1.07	1.05	1.06	1.07	1.02
70 (28 dpc)	0.9% saline	Geo Mean / Geo SD	N/A	N/A	N/A	N/A	N/A	N/A	N/A	N/A	N/A	N/A	N/A	N/A
	VSV-MARV vaccine	Geo Mean	8.22	3.49	0.11	2.97	0.95	0.07	6.13	11.45	38.28	62.45	18.72	29.94
		Geo SD	1.70	1.57	1.69	2.34	1.67	2.69	1.04	1.06	1.05	1.05	1.06	1.01

Day Post Vaccination	Group	Mean/SD	RDWc %	RDWs (fl)	PLT (10^9/L)	MPV (fl)	PCT %	PDWc %	PDWs (fl)	LYM %	MON %	NEU %	EOS %	BAS %
42 (0 dpc)	0.9% saline	Geo Mean	16.98	39.44	339.35	9.03	0.31	36.60	13.77	42.67	0.86	42.56	11.13	1.50
		Geo SD	1.03	1.03	1.38	1.02	1.38	1.03	1.05	1.22	1.33	1.24	1.07	1.00
	VSV-MARV vaccine	Geo Mean	17.20	40.71	173.50	9.70	0.17	38.40	16.79	40.50	1.78	40.83	9.56	1.32
		Geo SD	1.06	1.06	1.38	1.11	1.44	1.06	1.22	1.39	2.23	1.58	1.34	1.19
47 (5 dpc)	0.9% saline	Geo Mean	16.73	39.06	188.53	9.08	0.17	36.54	13.85	24.35	0.97	53.54	18.14	1.50
		Geo SD	1.03	1.02	1.41	1.02	1.44	1.01	1.05	1.32	1.35	1.18	1.11	1.00
	VSV-MARV vaccine	Geo Mean	17.34	41.30	186.84	9.85	0.18	38.45	16.63	42.25	0.92	41.29	11.54	1.48
		Geo SD	1.06	1.05	1.46	1.09	1.48	1.03	1.14	1.26	2.33	1.28	1.38	1.03
49 (7dpc, M)	0.9% saline	Geo Mean	17.36	41.66	186.99	9.94	0.18	39.82	17.55	61.56	5.15	16.00	15.73	1.50
		Geo SD	1.00	1.00	1.00	1.00	1.00	1.00	1.00	1.00	1.00	1.00	1.00	1.00
50 (8 dpc, M)	0.9% saline	Geo Mean	16.90	40.86	355.30	10.27	0.37	39.86	17.18	27.98	0.86	63.09	6.49	1.50
		Geo SD	1.00	1.00	1.00	1.00	1.00	1.00	1.00	1.00	1.00	1.00	1.00	1.00
52 (10 dpc)	0.9% saline	Geo Mean / Geo SD	N/A	N/A	N/A	N/A	N/A	N/A	N/A	N/A	N/A	N/A	N/A	N/A
	VSV-MARV vaccine	Geo Mean	17.28	41.13	284.45	10.02	0.28	38.54	16.86	49.30	1.28	30.06	14.69	1.50
		Geo SD	1.05	1.05	1.31	1.14	1.35	1.06	1.23	1.18	1.50	1.49	1.49	1.50
70 (28 dpc)	0.9% saline	Geo Mean / Geo SD	N/A	N/A	N/A	N/A	N/A	N/A	N/A	N/A	N/A	N/A	N/A	N/A
	VSV-MARV vaccine	Geo Mean	17.44	41.74	92.83	9.86	0.10	38.60	17.27	42.49	1.35	36.14	11.55	0.87
		Geo SD	1.06	1.06	4.58	1.15	3.80	1.09	1.30	1.67	1.84	1.49	1.23	3.36

Table S2. Summary statistics for CBC Experiment 2. N/A = NHP not on study (NHP deceased.) Data from d49/50 from one NHP. NS- no sample run. Abbreviations: Total white blood cell count (WBC), total lymphocyte cell count (LYM), total monocyte cell count (MON), total neutrophil cell count (NEU), total eosinophil cell count (EOS), total basophil cell count (BAS), total red blood cell count (RBC), total hemoglobin (HGB), hematocrit value (HCT), mean cell volume (MCV), mean corpuscular hemoglobin (MCHC), platelet count (PLT), mean platelet volume (MPV), plateletcrit (PCT), platelet distribution width (PDWc and PDWs), red cell distribution width (RDW), percent neutrophils (NEU%), percent lymphocytes (LYM%), precent monocytes (MON%), percent eosinophils (EOS%), percent basophils (BAS%).
